# Supplementary material for: Global hypo-methylation in a proportion of glioblastoma enriched for an astrocytic signature is associated with increased invasion and altered immune landscape
Source: eLife. 2022 Nov 22;11:e77335. doi: 10.7554/eLife.77335 (PMC9681209; doi:10.7554/eLife.77335)
Supplement: Figure 2—source data 1. [file elife-77335-fig2-data1.zip › Figure_2_source_data_1/Figure_2I_J/homerResults/motif2.similar.html]

motif2

## Information for motif2

A
G
T
C
C
G
T
A
A
T
C
G
G
T
A
C
G
C
T
A
A
T
G
C
A
C
G
T
A
C
T
G
  
Reverse Opposite:  

T
G
A
C
C
G
T
A
A
T
C
G
C
G
A
T
C
A
T
G
T
A
G
C
A
C
G
T
A
C
T
G
  

|  |  |
| --- | --- |
| p-value: | 1e-57 |
| log p-value: | -1.335e+02 |
| Information Content per bp: | 1.836 |
| Number of Target Sequences with motif | 59.0 |
| Percentage of Target Sequences with motif | 8.13% |
| Number of Background Sequences with motif | 1.6 |
| Percentage of Background Sequences with motif | 0.57% |
| Average Position of motif in Targets | 96.3 +/- 51.9bp |
| Average Position of motif in Background | 112.6 +/- 41.3bp |
| Strand Bias (log2 ratio + to - strand density) | 0.0 |
| Multiplicity (# of sites on avg that occur together) | 1.03 |
| Motif File: | file (matrix) reverse opposite |

### Similar de novo motifs found

|  |  |  |  |  |  |  |  |
| --- | --- | --- | --- | --- | --- | --- | --- |
| Rank | Match Score | Redundant Motif | P-value | log P-value | % of Targets | % of Background | Motif file |
| 1 | 0.687 | T A C G T C A G T A G C G C T A T A C G G T A C G T A C T G A C G A C T A T C G | 1e-36 | -84.508789 | 5.92% | 0.65% | motif file (matrix) |
| 2 | 0.780 | C G A T A G T C G C A T A T G C C G T A A T C G C G A T T C A G T G C A C G A T T C A G C G T A | 1e-32 | -75.478840 | 8.26% | 1.41% | motif file (matrix) |
| 3 | 0.603 | G T C A A C T G A G T C A G T C G T A C A C G T A C T G A C G T | 1e-30 | -70.008376 | 8.95% | 1.58% | motif file (matrix) |
| 4 | 0.692 | C T A G T A G C C G T A A T C G G T A C T A C G T A G C G C A T A C T G T G A C G T C A A T C G | 1e-20 | -46.790081 | 3.99% | 0.64% | motif file (matrix) |
| 5 | 0.602 | A T C G T C G A T A G C G C T A T G A C G T C A A G T C G T A C C G T A A C T G C T A G T A C G | 1e-14 | -32.596834 | 3.17% | 0.30% | motif file (matrix) |
